# ABA seed priming alleviates low-temperature-induced inhibition

**DOI:** 10.3389/fpls.2026.1866445

**Published:** 2026-07-09

**Authors:** Jiao Ren, Zexin Qi, Zhengwen Guan, Dongsheng Gai, Yutong Zhang, Jiehao Cui, Liying Guo, Yanqiu Geng, Qiang Zhang, Xiwen Shao

**Affiliations:** Agronomy College, Jilin Agricultural University, Changchun, Jilin, China

**Keywords:** abscisic acid, antioxidant, direct-seeded rice, low temperature, seed germination

## Abstract

**Introduction:**

Abscisic acid (ABA) is a key regulator of seed dormancy and stress responses, but its effects on seed germination depend on concentration, developmental stage, and environmental conditions. This study investigated whether exogenous ABA seed priming could alleviate low-temperature-induced inhibition of germination and improve early seedling establishment in direct-seeded rice.

**Methods:**

Rice seeds were primed with different ABA concentrations and then exposed to low-temperature stress during germination and early seedling growth. Germination performance, reserve mobilization, respiratory metabolism, antioxidant responses, endogenous ABA and GA contents, selected SOD-related gene expression, and seedling growth traits were analyzed.

**Results:**

ABA seed priming showed concentration-dependent effects, with 40 μmol L^-1^ ABA exhibiting the most favorable overall performance under low-temperature conditions. Compared with the low-temperature control, low-to-moderate ABA priming improved germination performance, enhanced α-amylase activity, increased soluble sugar accumulation, and increased respiratory rate. ABA priming also enhanced antioxidant enzyme activities and reduced H_2_O_2_ and malondialdehyde accumulation. Expression analysis of selected SOD-related genes showed gene-specific responses rather than uniform transcriptional activation. In addition, ABA seed priming was associated with lower endogenous ABA accumulation, higher GA content, and a reduced ABA/GA ratio under low-temperature stress. The beneficial effects observed during germination were partly maintained at the three-leaf stage, particularly in root growth, chlorophyll accumulation, and antioxidant capacity.

**Discussion:**

These results suggest that appropriate ABA seed priming can improve germination and early seedling establishment under low-temperature stress in direct-seeded rice, mainly through physiological adjustments involving reserve mobilization, oxidative damage mitigation, antioxidant responses, and endogenous hormonal status. Further studies involving GA metabolism genes, ABA signaling components, and additional mechanistic validation are needed.

## 1 Introduction

Rice (*Oryza sativa* L.) is an important food crop in China (Fairhurst and Dobermann, 2002). Northeast China is one of the major rice-growing regions in the country, contributing 16–20% of national rice production annually. Its abundant sunlight and favorable temperature resources support the production of high-quality rice (Yang et al., 2020), making stable yield improvement in this region important for national food security. However, rice production in Northeast China is increasingly threatened by multiple climatic stresses (Ju et al., 2013; Lv et al., 2018). Without adaptation measures, rice yield losses may reach approximately 8.9% for each 1 °C increase in growing-season temperature by the 2100s (Liu et al., 2020). Heat and cold stresses are also projected to become major constraints, causing yield reductions of 5% and 8%, respectively (Zhang et al., 2017). Moreover, climate change-induced rice yield losses could reach 44.9% by the 2080s (Wu et al., 2014). Low-temperature chilling injury frequently occurs in Chinese rice-growing regions, especially in Northeast China where late spring cold spells are common (Zhou et al., 2010; Liu et al., 2018). Therefore, improving germination performance and early seedling establishment under low-temperature conditions is essential for stable direct-seeded rice production. Low temperature significantly inhibits rice germination and seedling growth, resulting in reduced germination rate, weakened seedling vigor, abnormal development, and ultimately yield loss (Wang et al., 2024). Rice seeds germinate optimally at 25–35 °C and are highly sensitive to temperatures below 15 °C (Monajjem et al., 2023). During germination, starch mobilization provides soluble sugars and energy required for embryo growth. Low temperature suppresses amylolytic enzyme activity and delays reserve mobilization, thereby limiting energy supply for radicle protrusion (Fu et al., 2023). Low-temperature stress also weakens respiratory metabolism and decreases the energy supply required for seed germination. In addition, low temperature disrupts reactive oxygen species (ROS) homeostasis in seed cells and impairs the efficiency of the antioxidant defense system, leading to excessive ROS accumulation and cellular damage (Fu et al., 2025). Furthermore, chilling stress can accelerate chlorophyll degradation, inhibit chlorophyll biosynthesis, and eventually cause leaf wilting or even seedling death (Basit et al., 2024; Mai et al., 2024). Therefore, reserve mobilization, respiratory metabolism, antioxidant defense, and photosynthetic capacity are important physiological processes associated with rice tolerance to low-temperature stress during germination and early seedling growth.

Abscisic acid (ABA) is an important stress-related phytohormone involved in plant responses to various abiotic stresses. Exogenous ABA can enhance plant stress resistance through different application methods, including foliar spraying, root immersion, and seed priming. For example, exogenous ABA root immersion treatment enhanced the salt-alkali stress resistance of rice seedlings and improved seedling performance under stress conditions (Gurmani et al., 2013). Furthermore, studies have found that ABA also plays a significant role in rice under low-temperature stress (Mittler and Blumwald, 2015), inducing various physiological changes and transcriptional expression of genes, thereby regulating the ability to resist cold stress (Rubio et al., 2019). ABA signaling is mainly mediated by the PYR/PYL/RCAR–PP2C–SnRK2 module, which regulates downstream stress-responsive transcriptional networks. Recent studies have demonstrated that ABA-mediated regulation of seed germination and stress adaptation involves ABA biosynthesis, catabolism, perception, and signaling components, including NCED, CYP707A, PYL receptors, PP2Cs, SnRK2s, and ABI family transcription factors, which coordinate hormonal signaling with reserve mobilization and antioxidant defense systems (Li et al., 2022; Mo et al., 2024). These signaling events may enhance antioxidant defenses by regulating genes encoding superoxide dismutase, catalase, peroxidase, and related ROS scavenging components (Mo et al., 2024; Zhao et al., 2025).

Seed germination is tightly controlled by the antagonistic interaction between ABA and gibberellins (GA). ABA is generally recognized as a negative regulator of seed germination and a key hormone involved in dormancy maintenance and stress protection, whereas GA promotes endosperm weakening, α-amylase production, reserve mobilization, and embryo growth (Yang et al., 2019; Xiao et al., 2025). Therefore, the ABA/GA balance, rather than the absolute level of either hormone alone, is critical for determining whether seeds remain dormant or proceed toward germination. Under adverse environmental conditions, increased ABA accumulation may contribute to stress protection, but excessive or prolonged ABA signaling can inhibit germination and seedling growth. Thus, the interaction between ABA-associated stress adaptation and GA-associated growth regulation is central to seed germination under chilling conditions.

Although ABA is classically considered a germination-inhibiting hormone, its physiological effect is highly dependent on concentration, developmental stage, treatment method, and environmental context. Under non-stress conditions, excessive ABA usually suppresses germination by maintaining dormancy-related signaling. However, when applied as a seed priming agent before exposure to stress, appropriate ABA concentrations may improve stress preparedness, enhance membrane stability, strengthen antioxidant capacity, and support osmotic and metabolic adjustment. Therefore, the beneficial effect of ABA seed priming under low-temperature stress should not be interpreted as ABA generally promoting germination, but rather as a context-dependent alleviation of low-temperature-induced inhibition.

Moreover, ABA-mediated seed priming has been reported to improve seed germination vigor under stress conditions by enhancing reserve mobilization, stabilizing ROS homeostasis, altering hormonal status, and inducing physiological and molecular adjustments that may persist beyond germination, thereby supporting early seedling establishment (Li et al., 2022; Chen et al., 2023; Kim et al., 2025). However, it remains unclear whether ABA-induced priming effects established during germination persist during subsequent seedling development in direct-seeded rice. Although ABA has been widely implicated in seed germination, seed vigor, and abiotic stress responses through processes related to reserve mobilization, antioxidant metabolism, hormonal homeostasis, and ABA signaling pathways, the physiological basis by which exogenous ABA seed priming affects low-temperature tolerance during germination and early seedling establishment remains insufficiently understood (Li et al., 2022; Mo et al., 2024).

Therefore, this study investigated the effects of different ABA seed priming concentrations on storage reserve mobilization, energy metabolism, antioxidant responses, endogenous ABA and GA contents, and seedling growth under low-temperature stress. We hypothesized that appropriate ABA seed priming would alleviate low-temperature-induced inhibition of rice germination by improving reserve utilization, enhancing antioxidant capacity, reducing oxidative damage, and altering endogenous ABA–GA status, with possible effects extending into early seedling development. Because this study mainly combines physiological, biochemical, hormone-content, and selected gene-expression analyses, the results are interpreted as evidence for physiological associations rather than definitive proof of direct molecular regulation of GA metabolism or ABA signaling. This work provides a physiological basis for evaluating ABA seed priming as a potential strategy to improve cold-stressed germination and early seedling establishment of direct-seeded rice in cold regions.

## 2 Materials and methods

### 2.1 Test materials

The rice cultivar Changbai 9, a japonica rice cultivar developed by the Jilin Academy of Agricultural Sciences, was used in this study. Changbai 9 is a medium-maturing cultivar widely cultivated in Northeast China, particularly in Jilin Province and other cold rice-growing regions. Previous modeling studies have further highlighted the importance of understanding crop growth and biomass production in cold-region rice paddy systems (Gao et al., 2023). Previous studies have reported that Changbai 9 exhibits moderate sensitivity to low-temperature stress during germination and early seedling establishment, making it a suitable material for evaluating physiological responses to chilling conditions. Therefore, this cultivar was selected as the experimental material for investigating the effects of ABA seed priming under low-temperature stress under low-temperature stress (“Wang et al., 2013).

### 2.2 Experimental design

#### 2.2.1 Experimental overview and timeline

The experiment consisted of two sequential phases: a low-temperature germination phase and a subsequent seedling growth phase. Rice seeds were soaked in distilled water or ABA solutions at different concentrations at 25 ± 0.5 °C for 48 h. The ABA concentration range was selected based on preliminary experiments and previous studies on ABA-mediated stress regulation in rice and other crops, which demonstrated that low-to-moderate ABA concentrations could enhance stress tolerance, whereas excessive ABA may inhibit germination and seedling growth (Yu et al., 2023; Ahmed et al., 2026).

Seed germination assays were conducted according to the International Seed Testing Association (ISTA, 2022) standard procedures. After priming, seeds were placed in 9 cm Petri dishes lined with two layers of filter paper under controlled temperature conditions. Each treatment included three biological replicates, with 50 seeds per replicate. Seeds treated with distilled water (CT1) or ABA solutions (T1–T4) were subjected to low-temperature germination and growth tests at 15 ± 0.5 °C for 14 d, whereas distilled water-soaked seeds germinated at 25 ± 0.5 °C were used as the normal-temperature control (CT2). Physiological indices were measured daily from day 3 to day 7 of germination, while morphological traits were determined on days 7 and 14. The treatment design used in this study is summarized in **Table 1**. ABA stock solution (Sigma-Aldrich, St. Louis, MO, USA) was prepared according to the manufacturer’s instructions and diluted with distilled water to the required concentrations before use.

**Table 1:** Treatment design of ABA seed priming under low-temperature stress.

Treatment	ABA concentration (μmol/L)	Germination temperature (°C)	Experimental role
CT1	0	15 ± 0.5	Low-temperature control
T1	20	15 ± 0.5	ABA treatment
T2	40	15 ± 0.5	ABA treatment
T3	60	15 ± 0.5	ABA treatment
T4	80	15 ± 0.5	ABA treatment
CT2	0	25 ± 0.5	Normal-temperature control

At the one-leaf-one-heart stage, seedlings were transferred to culture boxes containing International Rice Research Institute nutrient solution, with 30 plants per box, and further cultivated in an artificial climate chamber under controlled environmental conditions. The low-temperature cultivation conditions are shown in **Supplementary Table 1**. Plants were harvested at the three-leaf stage for physiological and morphological analyses. Both germination and seedling experiments adopted a completely randomized design with three biological replicates per treatment. Representative images of seedlings at 7 days post‐germination (A) and at the three‐leaf stage (B) are shown in Supplemental **Supplementary Figure 1**.

### 2.3 Germination percentage, germination energy, germination index, and mean germination time

Seed germination was monitored daily during the first 7 days of incubation. Radicle and plumule lengths were measured using a digital vernier caliper, with ten germinating seeds randomly selected from each replicate. Under low-temperature conditions, a seed was considered germinated when the plumule protruded at least 1 mm from the seed coat (Wang et al., 2016; Monajjem et al., 2023).

On the seventh day after sowing, germination percentage (GP), germination energy (GE), germination index (GI), and mean germination time (MGT) were calculated according to standard seed germination evaluation methods (Krishnasamy and Seshu, 1989; Wang et al., 2016).


$$GP\;\left(\%\right)\;=\;\left(Number\;of\;germinated\;seeds\;within\;7\;days/Total\;number\;of\;seeds\;tested\right)\;\times \;100$$

$$GE\;\left(\%\right)\;=\;\left(Number\;of\;germinated\;seeds\;within\;the\;first\;3\;days/Total\;number\;of\;seeds\;tested\right)\;\times \;100$$

$$GI\;=\;\Sigma \left(Gt/Dt\right)$$

$$MGT\;=\;\Sigma \left(Gt\;\times \;Dt\right)\;/\;\Sigma Gt$$

where Gt represents the number of seeds germinated on day t and Dt represents the corresponding germination time (days).

### 2.4 Morphological indices, fresh weight, and dry weight of germinated seeds

Morphological characteristics and biomass accumulation were evaluated at 7 and 14 days after germination. Ten germinating seeds were randomly collected from each replicate. Coleoptiles, radicles, and residual seed tissues were separated, and their fresh weights were recorded immediately.

For dry weight determination, samples were initially heated at 105 °C for 30 min to terminate metabolic activity and subsequently dried at 80 °C to constant weight. At the three-leaf stage, ten seedlings from each replicate were randomly sampled for root length measurement and biomass determination. Shoots and roots were separated, and both fresh weight and dry weight were measured using the same procedure described above.

### 2.5 Germinated seed starch and soluble sugar

The contents of soluble sugars and starch in germinating seeds were determined according to the methods described by Zhang and Chen (2008). Soluble sugar content was measured using the anthrone-sulfuric acid colorimetric method, whereas starch content was determined by polarimetric analysis.

### 2.6 Extraction and determination of amylase

α-Amylase activity was determined according to the method of Zhang and Chen (2008) with minor modifications. Crude enzyme extracts were prepared from germinating seed tissues, and enzyme activity was quantified colorimetrically by measuring absorbance at 540 nm. α-Amylase activity was calculated based on a standard calibration curve and expressed on a fresh-weight basis.

### 2.7 Seed respiration rate

Seed respiration rate was measured according to the method described by Wang et al. (2016). Measurements were conducted daily at a fixed time from the third to the seventh day after germination. The respiration rate was expressed as CO_2_ release per unit seed mass per unit time.

### 2.8 Reactive oxygen species and antioxidant enzymes

Superoxide dismutase (SOD) activity was determined using the nitroblue tetrazolium (NBT) photochemical reduction method according to Li et al. (2019), with minor modifications. Fresh germinating seed tissues (0.5 g) were homogenized in ice-cold extraction buffer and centrifuged at 10, 000 × g for 20 min at 4 °C. The resulting supernatant was used as the crude enzyme extract. SOD activity was quantified by monitoring the inhibition of NBT photoreduction at 560 nm. One unit (U) of SOD activity was defined as the amount of enzyme required to inhibit 50% of NBT photoreduction.

Peroxidase (POD) activity was determined using the guaiacol oxidation method described by Kochba et al. (1977). The reaction mixture consisted of crude enzyme extract and freshly prepared guaiacol reaction solution. Changes in absorbance at 470 nm were recorded at 30-s intervals. One unit of POD activity was defined as an increase of 0.01 absorbance units per minute.

Malondialdehyde (MDA) content was measured using the thiobarbituric acid (TBA) method according to Li et al. (2019). Following reaction with TBA, absorbance values were recorded at 450, 532, and 600 nm, and MDA concentration was calculated using the corresponding formula.

Hydrogen peroxide (H_2_O_2_) content was determined using the potassium iodide method described by Zhang and Chen (2008), with minor modifications. Fresh germinating seed tissues (0.2 g) were homogenized in 0.1% trichloroacetic acid and centrifuged at 12, 000 × g for 15 min at 4 °C. The supernatant was subsequently reacted with potassium phosphate buffer and potassium iodide solution, and absorbance was measured at 390 nm. H_2_O_2_ concentration was calculated using a standard curve.

### 2.9 ABA and GA content

Endogenous abscisic acid (ABA) and gibberellin (GA) contents were determined using liquid chromatography–mass spectrometry (LC–MS/MS). Fresh germinating seed tissues (0.2 g) were ground into fine powder in liquid nitrogen under ice-bath conditions and extracted with 3 mL of 80% methanol. The homogenate was transferred into a 10 mL centrifuge tube and extracted on a shaker under ice-bath conditions for 12 h, followed by centrifugation at 12, 000 r/min for 15 min at 4 °C. The supernatant was collected, and the residue was re-extracted with an additional 3 mL of 80% methanol under the same conditions. The two supernatants were combined and used for subsequent analysis. ABA and GA contents were quantified using a Shimadzu LCMS-8050 liquid chromatography–mass spectrometry system (Shimadzu, Kyoto, Japan) (Lin et al., 2017).

### 2.10 RNA extraction and gene expression analysis

Analysis was performed using fluorescence-based real-time quantitative PCR, with the Q3 Real-time PCR instrument from Applied Biosystems (ABI) and SYBR Premix Ex TaqTM reagents from Takara Bio Engineering Co., Ltd. For gene expression analysis, whole germinating seed tissues were collected at 7 days after germination from each treatment. Approximately 0.1 g of fresh germinating seed tissue was immediately frozen in liquid nitrogen and ground to a fine powder using a pre-chilled mortar and pestle. Total RNA was extracted according to the manufacturer’s instructions. The quality and concentration of RNA extracted from germinating seed tissues were evaluated for absorbance at OD260 and OD280 using a spectrophotometer to assess RNA quality and determine RNA concentration. Qualified samples were stored at -80°C for future use. Add 4μL RNA (400ng), 2μL 5×gDNA Easer Buffer, and 1μL gDNA Easer into a PCR tube. Perform the operation on ice and mix well using a pipette tip. Incubate at 42°C for 2 minutes, then store at 4°C. For RNA reverse transcription, add 1μL PrimeScript RT Enzyme Mix I, 1μL RT Primer Mix, 4μL 5×PrimeScript Buffer 2, and 4μL RNase-Free dH2O to the reaction mixture from the previous step. Incubate at 37°C for 15 minutes, followed by a reaction at 85°C for 5 seconds. Real-Time PCR amplification was performed using the SYBR Green two-step method with a 20 μL reaction system. The reaction conditions were as follows: pre-denaturation at 95°C for 30 s; PCR reaction at 95°C for 5 s and 60°C for 34 s, for 40 cycles. The reverse-transcribed cDNA solution was diluted 20-fold, and the reaction was carried out according to the system shown in **Supplementary Table 2**. Specific operational steps followed the instructions of the Takara kit.

Relative gene expression levels were calculated using the 2^-△△CT^ method (Livak and Schmittgen, 2001). The rice *Actin-1* gene was used as the internal reference gene to normalize the expression levels of target genes. For each sample, ^ΔCT^ was calculated as the difference between the CT value of the target gene and that of *Actin-1*. The control treatment was used as the calibrator, and ^ΔΔCT^ was calculated by subtracting the ^ΔCT^ value of the control from that of each ABA treatment. The relative expression level was then expressed as 2^-ΔΔCT^. Primer sequences for all target genes and *Actin-1* are listed in **Supplementary Table 3**.

### 2.11 Chlorophyll content

Referring to the method of Zhang and Chen (2008), weigh 0.2 g of leaf tissue, grind it with 95% ethanol until the tissue turns white, and let it stand for 30 minutes. After filtration, dilute the solution to a 25 mL volumetric flask. Measure the absorbance at 665 nm, 649 nm, and 470 nm, and calculate the total chlorophyll content.

### 2.12 Data analysis

All experiments were conducted with three biological replicates, and the data are presented as mean ± standard deviation (SD). Statistical analyses were performed using SPSS 25.0 software (IBM Corp., Armonk, NY, USA), while figures were prepared using Excel 2019. Differences among treatments were analyzed by one-way analysis of variance (ANOVA) followed by Duncan’s multiple range test at the 0.05 significance level (*P*< 0.05).

## 3 Results and analysis

### 3.1 Effect of ABA seed priming on germination of direct-seeded rice

As shown in **Table 2**, with the increase of exogenous ABA concentration, the germination rate, germination potential, germination index and bud length of each treatment first increased and then decreased. Compared with CTI, T1, and T4 treatments, T2 treatment significantly improved germination rate, germination potential, and germination index. The germination rates increased by 5.19%, 4.80%, and 5.94% respectively; while the germination potentials improved by 16.19%, 25.40%, and 15.00% respectively. The germination index increased by 11.64%, 9.11%, and 13.50% respectively. with the increase of exogenous ABA concentration, the seed dry weight showed a trend of first decreasing and then increasing. Compared with CT1, T2 and T3 significantly reduced the residual seed dry weight by 8.79% and 6.98% respectively. The shoot dry weight initially increased and then decreased, with no significant differences observed among treatments. Exogenous ABA significantly affected the mean germination time in all cases.

**Table 2:** Effects of pre-soaked with exogenous ABA on germination in direct-seeded rice.

Treatment	Germination percentage (%)	Germination energy (%)	Germination index	Mean germination time (d)	Length (mm)	Dry weight (mg seed^-1^)	
					Shoot	Shoot	Residual seed
T1	93.67 ± 2.08b	42.00 ± 5.29c	42.38 ± 0.44c	5.24 ± 0.02a	5.09 ± 0.34b	0.15 ± 0.02b	26.33 ± 0.33a
T2	98.17 ± 1.53a	52.67 ± 1.15b	46.24 ± 0.06b	5.18 ± 0.02a	6.19 ± 0.33b	0.20 ± 0.01b	24.69 ± 0.12b
T3	96.67 ± 3.06ab	43.33 ± 21.01c	43.24 ± 2.80bc	5.26 ± 0.11a	5.83 ± 0.17b	0.19 ± 0.06b	25.18 ± 0.55b
T4	92.67 ± 1.15bc	37.67 ± 6.43c	40.74 ± 1.14c	5.28 ± 0.04a	4.92 ± 0.40b	0.17 ± 0.04b	26.28 ± 0.12a
CT1	93.33 ± 1.16b	45.33 ± 4.16c	41.42 ± 1.63c	5.27 ± 0.05a	5.17 ± 0.48b	0.16 ± 0.05b	27.07 ± 0.51a
CT2	98.67 ± 1.21a	97.33 ± 3.06a	82.36 ± 3.50a	4.46 ± 0.04b	22.30 ± 1.11a	1.50 ± 0.04a	22.15 ± 0.96c

Different small letters in the same column refer to significant difference between treatments at *P*<0.05 level.

### 3.2 Effects of exogenous ABA soaking on starch content in germinating seeds of direct-seeded rice

**Figure 1:**
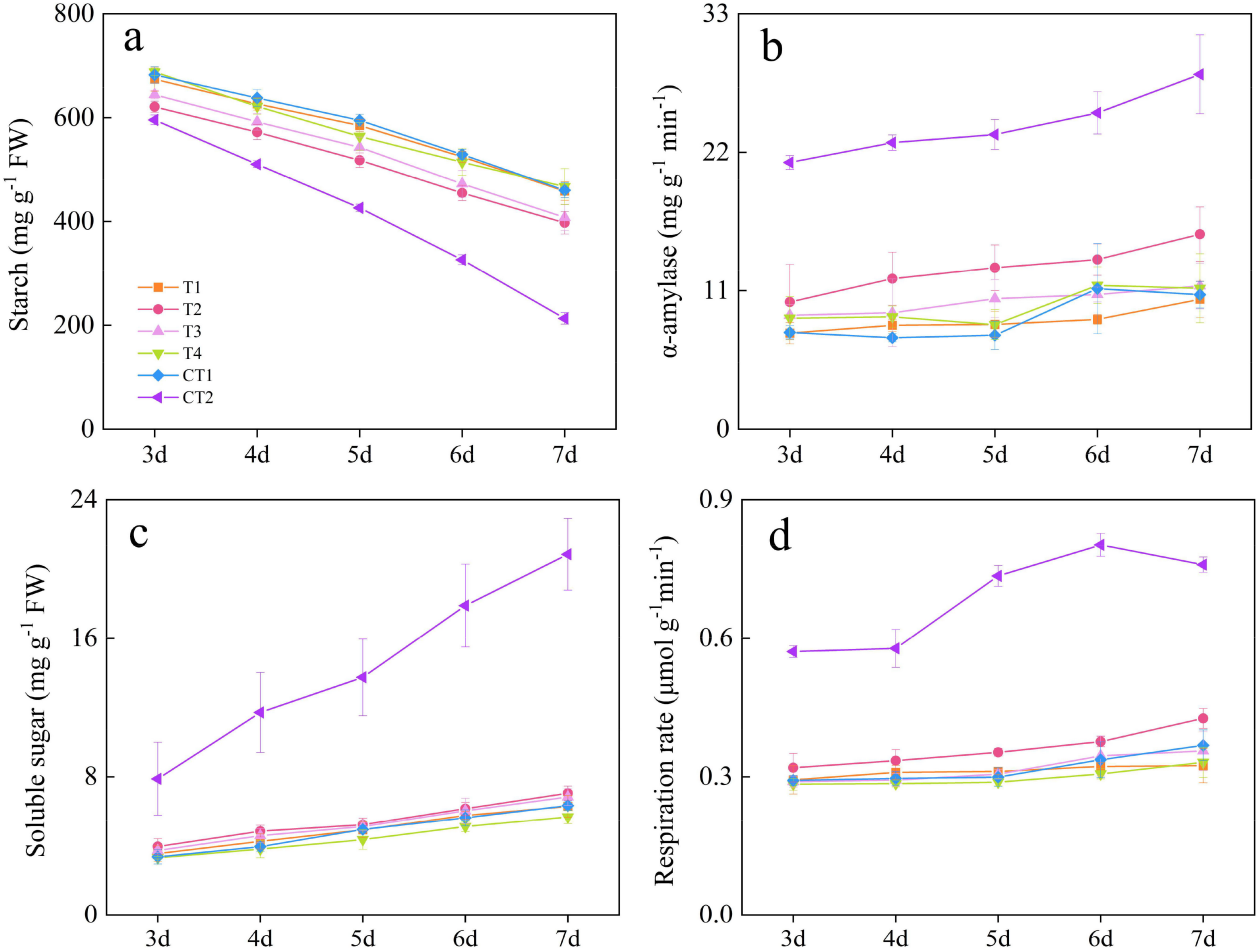
Effects of ABA seed priming on physiological indices during germination of direct-seeded rice. **(a)** starch content. **(b)** α-amylase activity. **(c)** soluble sugar content. **(d)** respiratory rate. Different small letters in the same column refer to significant difference between treatments at *P*<0.05 level. The following figures are the same.

As shown in **Figure 1a**, the starch content of all treatments decreased with increasing germination time, with the CT2 treatment showing significantly lower starch content than other treatments. As the concentration of exogenous ABA increased, the starch content of each treatment exhibited an initial decrease followed by an upward trend. During the 3–7 days of germination, the starch content in T2 and T3 treatments showed a significant decrease compared to CT1 treatment. On the 3rd day of germination, a significant decrease of 8.97% and 5.56% was observed. On the 4th day, a significant decrease of 10.31% and 7.18%. On the 5th day, a significant decrease of 12.96% and 8.76%. On the 6th day, a significant decrease of 14.02% and 10.70%; and on the 7th day, a significant decrease of 13.64% and 11.36%. During days 3–7 of germination, there was no significant difference between T1 and T4 treatments compared to CT1 treatment.

### 3.3 Effect of exogenous ABA soaking on amylase activity in germinating seeds of direct-seeded rice

As shown in **Figure 1b**, α-amylase activity exhibited a concentration-dependent response to exogenous ABA treatment, increasing initially and then decreasing with increasing ABA concentration. Compared with CT1, low-to-moderate ABA treatments generally enhanced α-amylase activity during germination under low-temperature stress, with T2 showing the strongest stimulatory effect. Significant increases were observed in T2 on days 4, 5, and 7, reaching 64.71%, 72.02%, and 45.18% above CT1, respectively. T3 also significantly increased α-amylase activity on day 5, whereas T4 showed a significant increase only on day 6. These results indicate that appropriate ABA concentrations promoted starch degradation by enhancing α-amylase activity, thereby facilitating reserve mobilization during germination under low-temperature conditions.

### 3.4 Effects of exogenous ABA soaking on soluble sugar content in germinating seeds of direct-seeded rice

As shown in **Figure 1c**, soluble sugar content also displayed a concentration-dependent pattern, increasing initially and then decreasing as ABA concentration increased. Compared with CT1, T2 and T3 significantly increased soluble sugar content on day 4 by 22.78% and 16.20%, respectively. On day 7, T2 maintained the highest soluble sugar accumulation, remaining significantly higher than CT1 by 11.37%. Overall, low-to-moderate ABA treatments promoted soluble sugar accumulation during germination, with T2 exhibiting the most pronounced effect.

### 3.5 Effect of exogenous ABA soaking on respiration rate of direct-seeded rice during germination stage

As shown in **Figure 1d**, seed respiration rate displayed a concentration-dependent response to ABA treatment, increasing initially and then decreasing with increasing ABA concentration. T2 consistently showed the highest respiration rate and significantly increased respiration by 10.00–16.67% relative to CT1 during the middle and late stages of germination. In contrast, the stimulatory effects of T1 and T3 were less pronounced, whereas T4 showed little or no improvement. These results suggest that low-to-moderate ABA concentrations enhanced respiratory metabolism under low-temperature stress, with T2 exhibiting the strongest effect. The increased respiration rate corresponded closely with the enhanced α-amylase activity and soluble sugar accumulation observed in the same treatment.

### 3.6 Effects of exogenous ABA soaking on the antioxidant system and related gene expression during germination of direct-seeded rice

**Figure 2:**
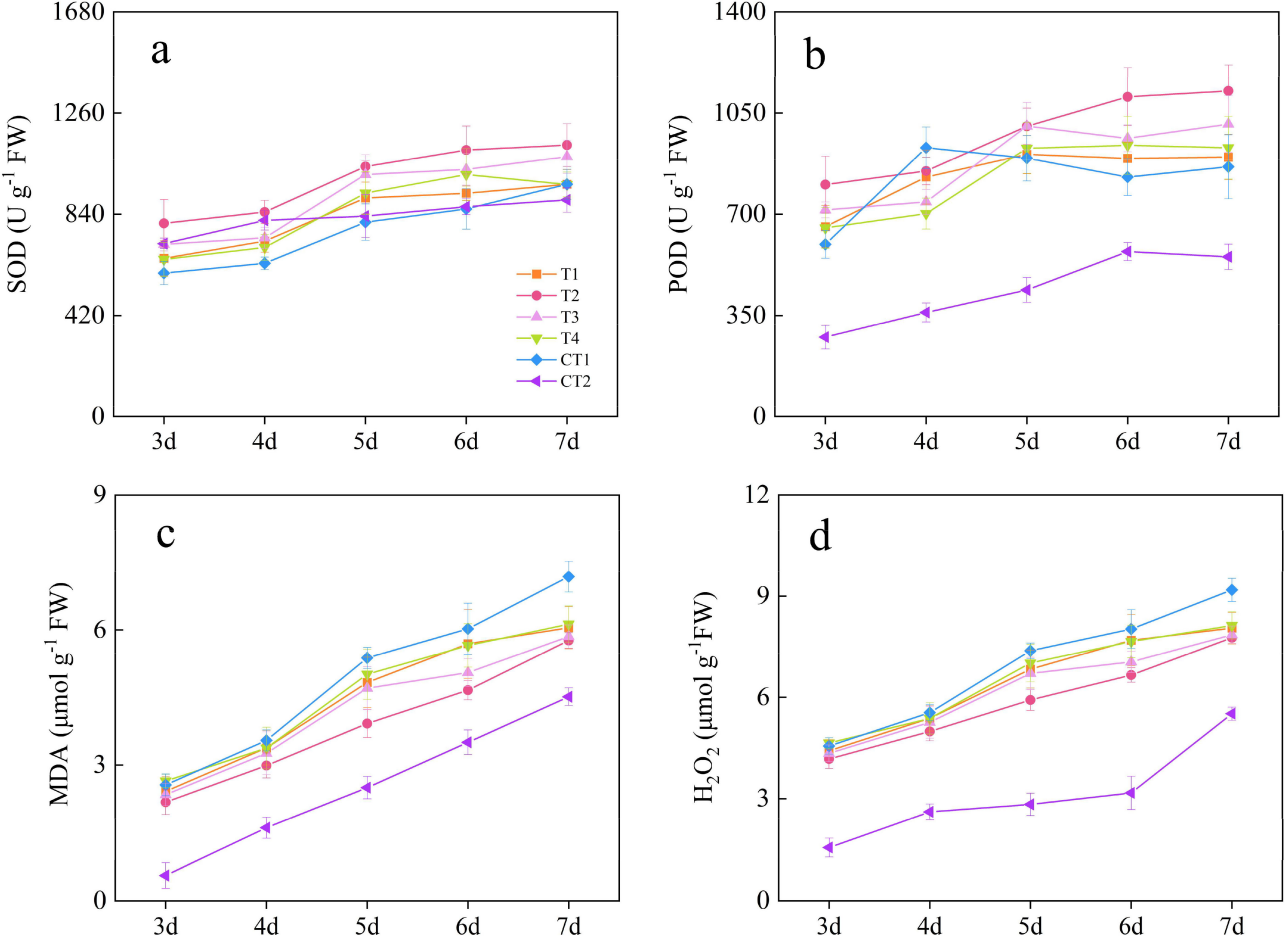
Effects of ABA seed priming on antioxidant indices during germination of direct-seeded rice. **(a)** SOD activity. **(b)** POD activity. **(c)** MDA content. **(d)** H_2_O_2_ content.

As shown in **Figure 2a**, SOD activity initially increased and then decreased with increasing ABA concentration. Compared with CT1, T2 consistently exhibited the highest SOD activity throughout germination and significantly increased SOD activity by 34.61%, 33.44%, 28.60%, 28.38%, and 16.90% on days 3–7, respectively. T3 also significantly enhanced SOD activity during most sampling periods, although the magnitude of increase was generally lower than that observed in T2. Compared with CT2, both T2 and T3 maintained significantly higher SOD activity during the middle and late stages of germination. These results indicate that low-temperature stress reduced antioxidant capacity, whereas appropriate ABA concentrations effectively enhanced SOD activity, thereby strengthening antioxidant defense during rice seed germination.

As shown in **Figure 2b**, POD activity showed a concentration-dependent pattern, increasing initially and then declining with increasing ABA concentration. Low-to-moderate ABA treatments generally enhanced POD activity under low-temperature stress, whereas the highest ABA concentration produced a weaker response. T2 exhibited the greatest enhancement and significantly increased POD activity by 33.54% and 30.43% relative to CT1 on days 6 and 7, respectively. T3 also promoted POD activity but to a lesser extent. Overall, ABA treatment alleviated the inhibitory effects of low temperature on antioxidant metabolism, with T2 showing the strongest stimulatory effect.

As shown in **Figure 2c**, low-temperature stress markedly increased MDA accumulation compared with CT2, indicating enhanced membrane lipid peroxidation under chilling conditions. Exogenous ABA treatment exhibited a concentration-dependent effect on MDA content, which initially decreased and then increased with increasing ABA concentration. Among all treatments, T2 consistently showed the lowest MDA content and significantly reduced MDA accumulation by 15.70–27.11% relative to CT1 during germination. T3 also effectively decreased MDA content, particularly during the later stages of germination. By day 7, all ABA treatments significantly reduced MDA content compared with CT1. Overall, exogenous ABA alleviated membrane oxidative damage under low-temperature stress, with the strongest effect observed in T2. The reduction in MDA content corresponded well with the enhanced antioxidant enzyme activities observed in ABA-treated seeds.

As shown in **Figure 2d**, H_2_O_2_ content was significantly higher under low-temperature stress than under normal temperature conditions (CT2), indicating increased oxidative stress during germination. Exogenous ABA treatment resulted in a concentration-dependent reduction in H_2_O_2_ accumulation, with the lowest levels observed under T2. Compared with CT1, T2 significantly reduced H_2_O_2_ content by 10.04–19.78% during the middle stages of germination and maintained the greatest reduction throughout the experimental period. T3 also effectively decreased H_2_O_2_ accumulation, although to a lesser extent than T2. By day 7, all ABA treatments significantly lowered H_2_O_2_ content relative to CT1. Overall, ABA treatment effectively mitigated oxidative stress induced by low temperature, and the variation pattern of H_2_O_2_ closely paralleled that of MDA content.

**Figure 3:**
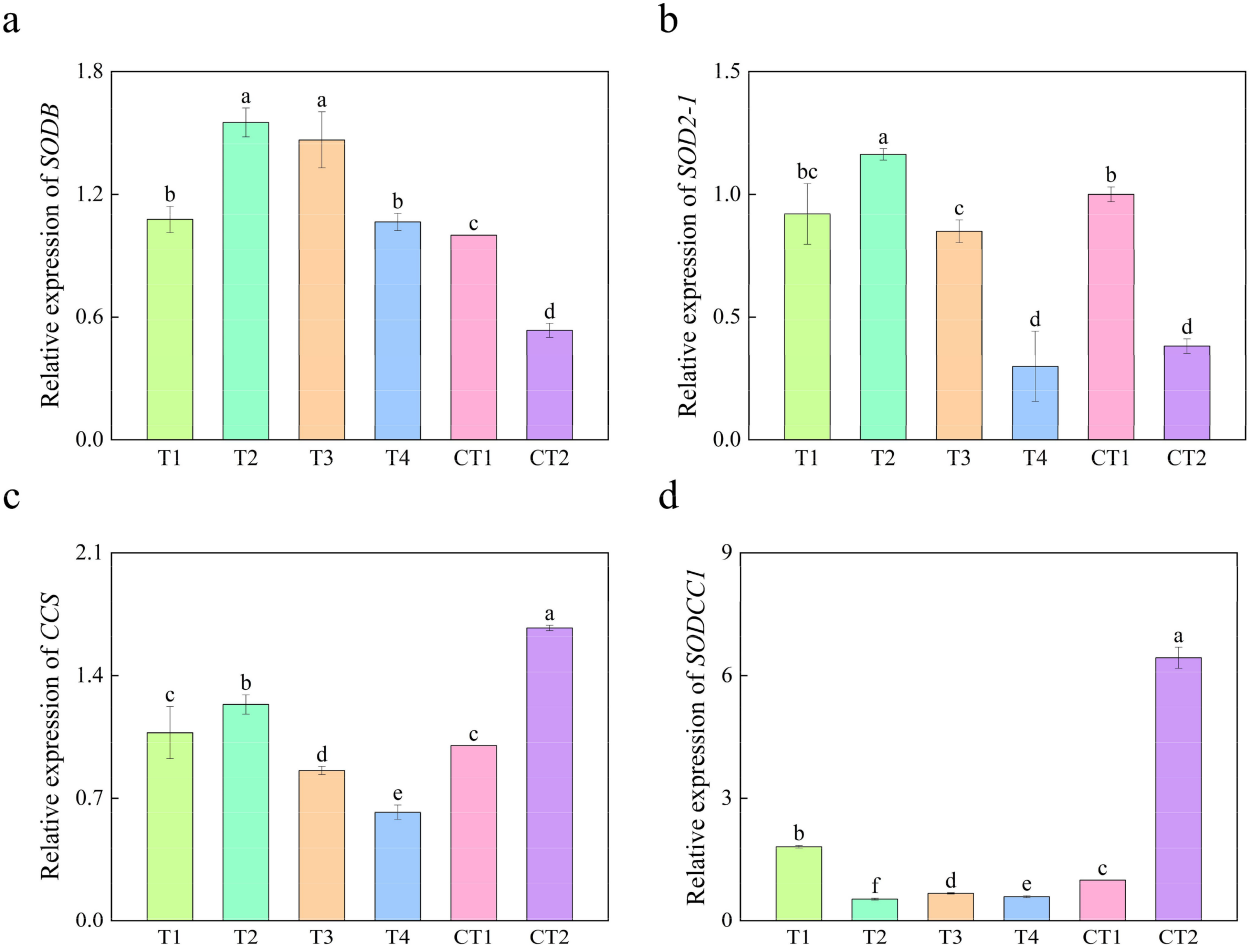
Effects of pre-soaking with exogenous ABA on the expression of antioxidant enzyme-related genes at 7 days of germination in direct-seeded rice. **(a)** *SODB*. **(B)** *SOD2-1*. **(C)** *CCS*. **(d)** *SODCC1*.

As shown in **Figure 3**, the relative expression levels of the examined SOD-related genes differed significantly among treatments. Compared with CT1, *SODB* expression was significantly increased by 55.14% and 46.62% in T2 and T3, respectively. *SOD2–1* expression was also significantly enhanced in T2, showing a 16.24% increase compared with CT1, whereas T3 and T4 showed lower expression levels than CT1. For *CCS*, T2 significantly increased its expression by 23.50% compared with CT1, while T3 and T4 reduced *CCS* expression. *SODCC1* expression was significantly increased by 81.33% in T1 compared with CT1, but decreased under T2, T3, and T4 treatments. Overall, ABA seed priming altered the expression of selected SOD-related genes in a concentration- and gene-specific manner. T2 enhanced the expression of *SODB*, *SOD2-1*, and *CCS*, whereas *SODCC1* was downregulated under T2, T3, and T4. These results indicate that ABA seed priming did not uniformly upregulate all examined SOD-related genes, but differentially affected specific components of the SOD-associated antioxidant system.

### 3.7 Effects of exogenous ABA soaking on endogenous abscisic acid and gibberellin during germination of direct-seeded rice

**Figure 4:**
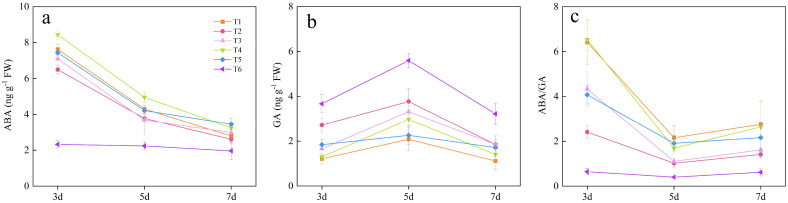
Effects of pre-soaking with exogenous ABA on endogenous ABA and GA contents in direct-seeded rice during germination. **(a)** ABA content. **(b)** GA content. **(c)** ABA/GA ratio.

As shown in **Figure 4**, low-temperature stress disrupted endogenous hormone homeostasis by decreasing GA content, increasing ABA content, and consequently elevating the ABA/GA ratio. During germination, endogenous ABA content generally declined, whereas GA content increased during the early stage and subsequently decreased. Exogenous ABA treatments exhibited a concentration-dependent effect on hormone dynamics, with low-to-moderate concentrations reducing endogenous ABA accumulation while promoting GA synthesis. Among all treatments, T2 showed the most pronounced response, significantly decreasing ABA content by 10.45–24.51% and increasing GA content by 47.64–66.67% relative to CT1. As a consequence, the ABA/GA ratio was markedly reduced, with T2 decreasing this ratio by 34.58–46.51% during germination. T3 produced similar but less pronounced effects. Overall, low-to-moderate ABA treatments, particularly T2, promoted a hormonal balance characterized by lower ABA levels, higher GA levels, and a reduced ABA/GA ratio under low-temperature stress.

### 3.8 Effects of exogenous ABA soaking on bud growth of direct-seeded rice

As shown in **Table 3**, exogenous ABA treatment promoted seedling growth and biomass accumulation while accelerating reserve mobilization in germinating seeds under low-temperature stress. Compared with CT1, low-to-moderate ABA treatments, particularly T2 and T3, significantly enhanced bud and root development. Among all treatments, T2 produced the strongest response, increasing bud dry weight, bud fresh weight, and bud length by 39.29%, 42.59%, and 47.31%, respectively. Root growth was also markedly promoted, with root dry weight, fresh weight, and length increasing by 96.15%, 88.15%, and 71.25%, respectively, compared with CT1. Similar but less pronounced improvements were observed in T3. In contrast, the dry and fresh weights of residual seeds were significantly reduced in T2 and T3, indicating enhanced mobilization and utilization of stored reserves during germination. Overall, appropriate ABA concentrations promoted seedling establishment by stimulating growth and reserve utilization, with T2 showing the most favorable effect.

**Table 3:** Effects of pre-soaked with exogenous ABA on growth of direct-seeded rice at bud stage.

Treatment	Shoot DW (mg)	Root DW (mg)	Seed DW (mg)	Shoot length (mm)	Root length (mm)	Shoot FW (mg)	Root FW (mg)	Seed FW (mg)
T1	1.02 ± 0.10bc	0.25 ± 0.05c	23.03 ± 1.46ab	16.67 ± 1.71bc	19.58 ± 5.24cd	6.63 ± 0.49b	2.90 ± 1.84bc	35.33 ± 1.48a
T2	1.17 ± 0.06b	0.51 ± 0.06b	19.11 ± 0.92c	19.96 ± 3.32b	27.52 ± 4.62b	7.60 ± 0.92b	5.40 ± 1.47b	31.20 ± 0.36b
T3	1.15 ± 0.06b	0.43 ± 0.03b	20.84 ± 1.13c	17.20 ± 0.67b	22.43 ± 2.95bc	7.07 ± 0.23b	4.57 ± 0.78b	36.93 ± 1.75a
T4	1.01 ± 0.09bc	0.42 ± 0.03b	21.02 ± 1.69bc	16.23 ± 1.12bc	21.57 ± 1.13bcd	6.73 ± 0.29b	4.10 ± 0.23b	35.47 ± 1.08a
CT1	0.84 ± 0.08c	0.26 ± 0.07c	24.05 ± 0.73a	13.55 ± 1.64c	16.07 ± 1.53d	5.33 ± 0.45c	2.87 ± 0.15c	37.40 ± 0.85a
CT2	4.14 ± 0.38a	2.34 ± 0.40a	11.70 ± 0.33d	45.94 ± 5.58a	40.10 ± 1.66a	22.57 ± 3.50a	11.57 ± 1.99a	31.57 ± 1.86b

Different small letters in the same column refer to significant difference between treatments at *P*<0.05 level.

### 3.9 Effects of ABA seed priming on the growth of three-leaf stage seedlings in direct-seeded rice

As shown in **Table 4**, low-temperature stress inhibited the growth of direct-seeded rice seedlings at the three-leaf stage, whereas exogenous ABA treatment promoted root development and biomass accumulation. Compared with CT1, T2–T4 significantly increased root length by 41.25–51.93%, root dry weight by 39.21–47.38%, and root fresh weight by 39.22–47.39%, with T4 showing the greatest enhancement. In contrast, higher ABA concentrations inhibited shoot growth, as reflected by significant reductions in shoot dry weight in T3 and T4 and shoot fresh weight in T4. Chlorophyll content was also increased by ABA treatment, with T2 and T3 showing significant increases of 14.18% and 9.11%, respectively. Overall, exogenous ABA improved seedling performance under low-temperature stress primarily by stimulating root growth and enhancing chlorophyll accumulation, although excessive ABA levels negatively affected shoot growth.

**Table 4:** Effects of pre-soaked with exogenous ABA on growth of direct-seeded rice at three leaf stage.

Treatment	Root length (cm)	Shoot DW (mg plant^-1^)	Root DW (mg plant^-1^)	Shoot FW (mg plant^-1^)	Root FW (mg plant^-1^)	Chlorophyll content (mg·g^-1^ FW)
T1	6.91 ± 0.77c	63.33 ± 1.76bc	14.86 ± 1.40c	324.65 ± 8.81b	104.00 ± 9.83c	3.93 ± 0.23c
T2	9.52 ± 1.12b	62.98 ± 1.44bc	20.45 ± 0.68b	324.57 ± 8.16b	143.17 ± 4.77b	4.51 ± 0.18b
T3	9.76 ± 1.85b	61.55 ± 1.97c	21.06 ± 1.89b	322.47 ± 9.19b	147.40 ± 13.26b	4.31 ± 0.11b
T4	10.24 ± 0.88b	60.67 ± 1.88c	21.65 ± 2.49b	306.02 ± 5.27c	151.57 ± 13.40b	4.05 ± 0.08c
CT1	6.74 ± 0.56c	64.96 ± 3.35b	14.69 ± 1.53c	335.79 ± 10.74b	102.85 ± 10.69c	3.95 ± 0.12c
CT2	13.10 ± 1.66a	86.40 ± 2.64a	37.76 ± 0.88a	432.02 ± 13.19a	224.32 ± 6.13a	5.70 ± 0.29a

Different small letters in the same column refer to significant difference between treatments at *P*<0.05 level.

### 3.10 Effects of exogenous ABA soaking on antioxidant enzymes in direct-seeded rice at three-leaf stage

**Figure 5:**
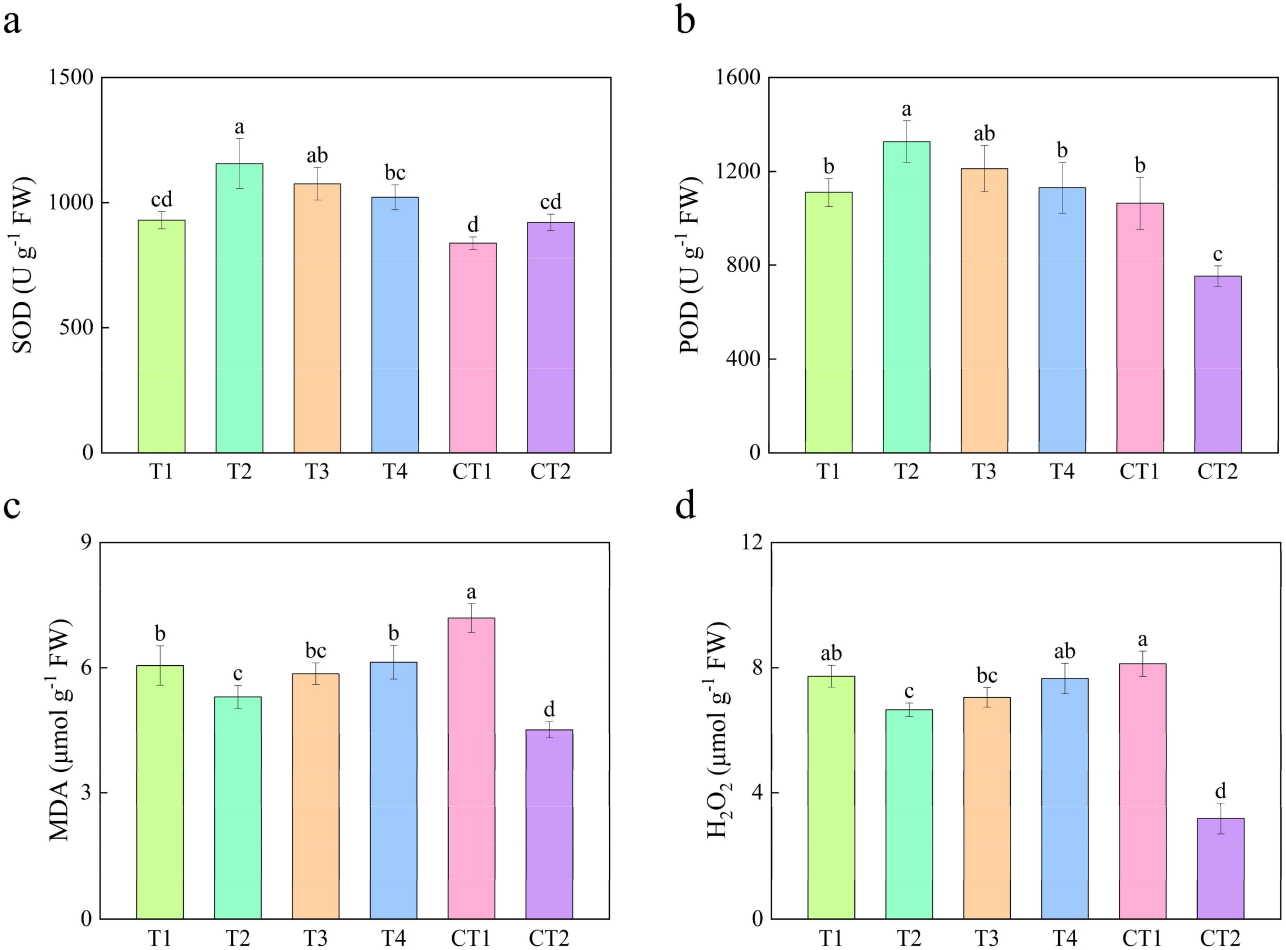
Effects of pre-soaking with exogenous ABA on antioxidant indices in direct-seeded rice at the three-leaf stage. **(a)** SOD activity. **(b)** POD activity. **(c)** MDA content. **(d)** H_2_O_2_ content.

As shown in **Figure 5**, low-temperature stress impaired the antioxidant defense system, as evidenced by reduced SOD and POD activities and increased MDA and H_2_O_2_ accumulation. Exogenous ABA treatment alleviated these adverse effects by enhancing antioxidant enzyme activities and reducing oxidative damage. Among all treatments, T2 exhibited the strongest response, significantly increasing SOD and POD activities by 37.91% and 24.71%, respectively, compared with CT1. In addition, T2 markedly reduced MDA and H_2_O_2_ contents by 26.25% and 18.04%, respectively. Similar but less pronounced effects were observed in T3 and T4. Overall, exogenous ABA enhanced antioxidant capacity and mitigated oxidative stress at the three-leaf stage under low-temperature conditions, with T2 showing the most favorable effect.

### 3.11 Correlation analysis between germination stage and seedling stage responses

**Figure 6:**
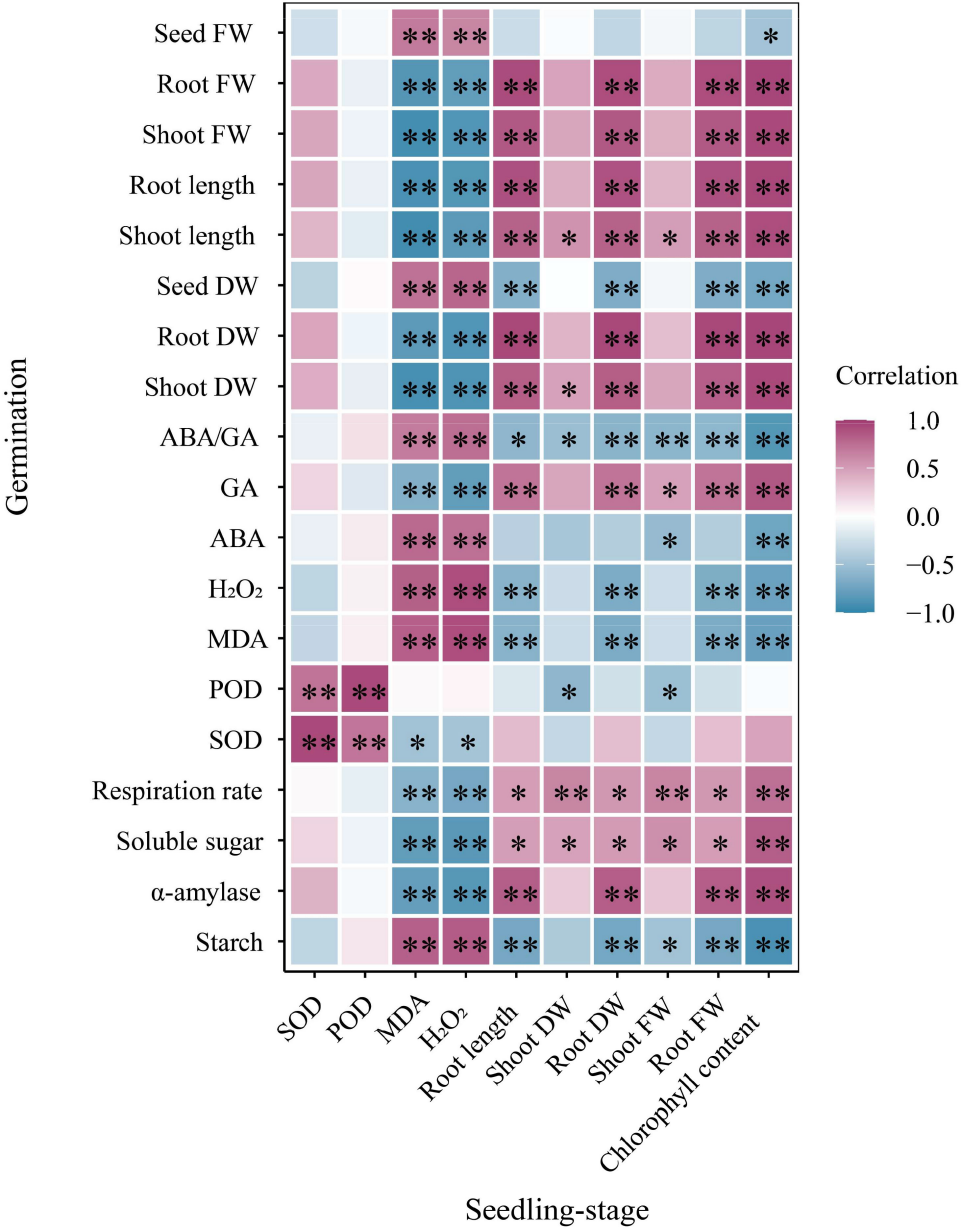
Pearson correlation heatmap between germination-stage physiological responses and seedling-stage growth traits under ABA seed priming treatments. Red and blue colors indicate positive and negative correlations, respectively. Asterisks indicate significant correlations (*P< 0.05, **P< 0.01).

To further clarify the relationship between ABA-induced responses during germination and subsequent seedling growth, Pearson correlation analysis was performed among germination-stage physiological parameters and seedling-stage growth and stress-response traits. As shown in **Figure 6**, germination-related traits, α-amylase activity, soluble sugar content, and antioxidant enzyme activities during germination were positively correlated with seedling growth parameters, especially root growth and seedling biomass. In contrast, MDA content, H_2_O_2_ content, and the ABA/GA ratio were negatively correlated with seedling growth traits. These results suggest that ABA priming effects initiated during germination may contribute to later seedling growth advantages by improving reserve mobilization, reducing oxidative damage, and regulating endogenous hormone balance.

### 3.12 Concentration-dependent trends of ABA seed priming under low-temperature stress

**Figure 7:**
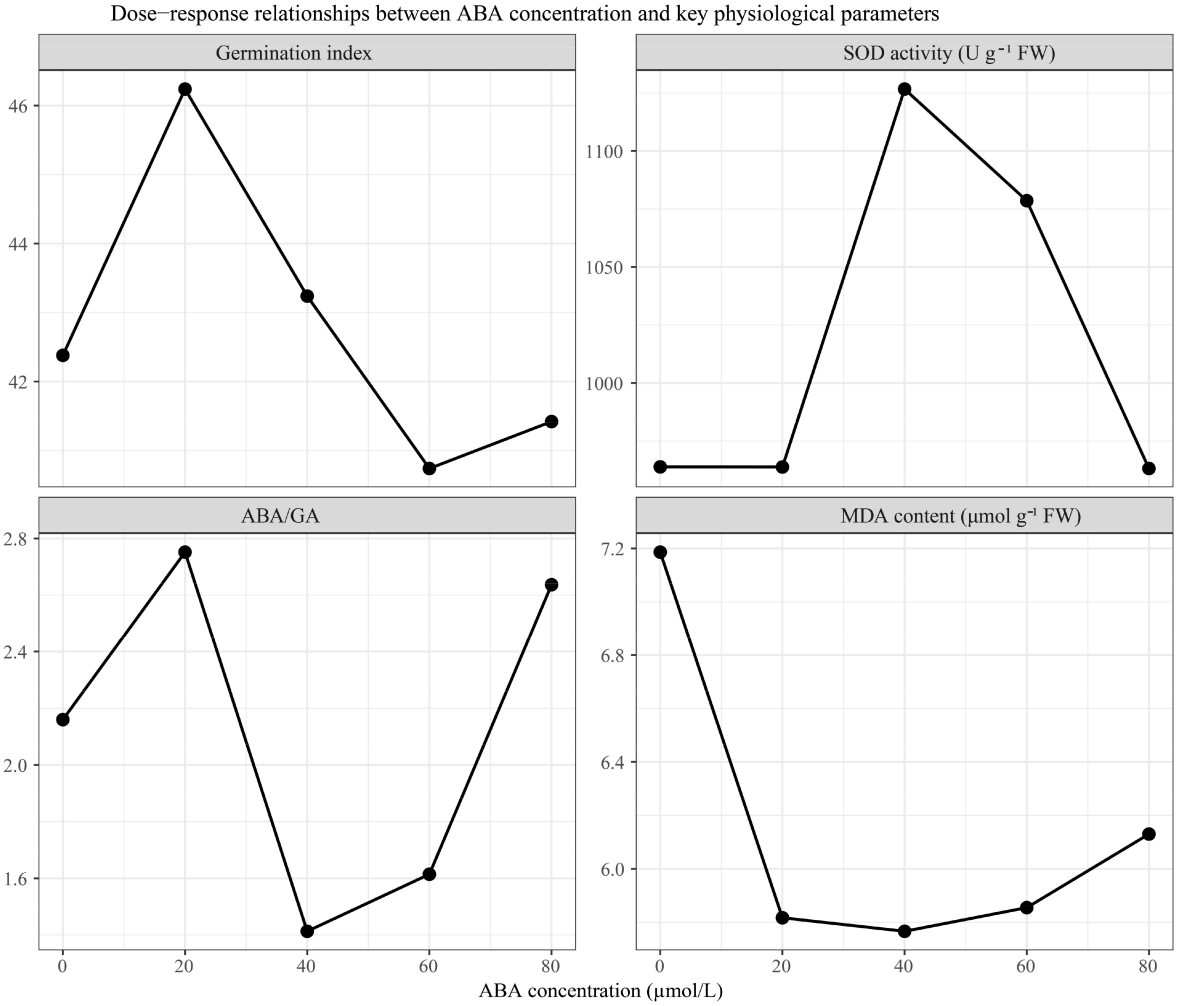
Dose-response relationships between exogenous ABA concentrations and representative physiological parameters under low-temperature stress. Values from the final germination stage, day 7, were used for analysis. The parameters shown are germination index, SOD activity, ABA/GA ratio, and MDA content.

To further evaluate the concentration-dependent effects of ABA seed priming under low-temperature stress, dose-response curves were constructed using representative physiological parameters (**Figure 7**). The responses of germination index, SOD activity, ABA/GA ratio, and MDA content exhibited clear concentration-dependent patterns under different ABA treatments. Low-to-moderate ABA concentrations improved germination performance and antioxidant capacity, whereas excessive ABA concentrations showed inhibitory effects. Among all treatments, 40 μmol/L ABA (T2) consistently exhibited the most favorable response, including higher germination index and SOD activity, together with lower ABA/GA ratio and MDA accumulation. By contrast, higher ABA concentration (80 μmol/L) weakened these beneficial effects. These results indicate that the physiological responses of direct-seeded rice to exogenous ABA under low-temperature stress are dose-dependent, and that appropriate ABA concentration is critical for balancing stress adaptation and growth regulation.

## 4 Discussion

### 4.1 ABA seed priming is associated with improved reserve mobilization and energy supply during cold-stressed germination

The primary energy and nutrients required for rice seed germination are derived from storage reserves in the endosperm, with starch accounting for a large proportion of seed dry weight (Esquivel-Fajardo et al., 2025). Alpha-amylase is a key enzyme involved in starch hydrolysis; however, its activity is highly temperature-sensitive and can be markedly reduced under low-temperature stress, leading to insufficient starch degradation and delayed germination. Previous studies have shown that alpha-amylase activity is positively associated with respiratory rate, germination performance, seedling quality, and biomass accumulation in rice (Cui et al., 2002; Krishnasamy and Seshu, 1989). Soluble sugars produced from starch degradation serve as respiratory substrates, while respiratory metabolism provides energy for coleoptile elongation and radicle protrusion (Monajjem et al., 2023). Low temperature suppresses respiratory metabolism, downregulates key metabolites in the EMP pathway and TCA cycle, and reduces energy supply during germination (Liu et al., 2018; Wang et al., 2016). Therefore, maintaining active reserve mobilization and respiration is important for successful germination under chilling conditions.

In this study, low-temperature stress reduced alpha-amylase activity, inhibited starch degradation, and decreased respiratory rate, thereby suppressing seed germination. In contrast, low-to-moderate concentrations of ABA seed priming increased alpha-amylase activity, reduced starch content and residual seed dry matter, and enhanced soluble sugar content and respiratory rate under low-temperature stress (**Figure 1**). These physiological changes were accompanied by increased germination percentage, germination energy, and germination index, as well as improved shoot and root growth. The increased soluble sugar content may have provided additional respiratory substrates, thereby supporting respiratory metabolism and early seedling growth under low-temperature stress.

The association between ABA seed priming and reserve mobilization may also be related to hormonal status. GA-mediated induction of alpha-amylase synthesis is a key process controlling starch degradation and seed germination (Park et al., 2010). Although ABA is traditionally regarded as a germination-inhibiting hormone, appropriate ABA seed priming may enhance stress preparedness and help maintain metabolic activity under adverse conditions. In the present study, increased alpha-amylase activity and soluble sugar accumulation under T2 and T3 treatments coincided with increased GA content and a reduced ABA/GA ratio. These results suggest that ABA seed priming was associated with improved reserve mobilization under low-temperature stress. However, because GA metabolism-related genes were not examined, the present data cannot determine whether ABA seed priming directly regulated GA biosynthesis or catabolism. Similar associations among hormonal status, carbohydrate metabolism, and germination performance have been reported in rice under chilling conditions (Monajjem et al., 2023; Wang et al., 2016).

### 4.2 ABA seed priming improves antioxidant enzyme activities and alleviates oxidative damage

Low-temperature stress disrupts cellular redox homeostasis and induces excessive accumulation of reactive oxygen species (ROS), such as H_2_O_2_ and superoxide radicals. Excessive ROS can trigger membrane lipid peroxidation, resulting in MDA accumulation and damage to cellular membrane systems (Song et al., 2021). Severe ROS accumulation may further interfere with normal cellular metabolism and eventually lead to cell death (Liu et al., 2025). Therefore, maintaining ROS homeostasis is an important protective mechanism for rice under low-temperature stress.

Antioxidant enzymes, including SOD and POD, play central roles in ROS scavenging. SOD catalyzes the conversion of superoxide radicals into H_2_O_2_, whereas POD participates in the subsequent detoxification of H_2_O_2_. Previous studies have demonstrated that ABA-dependent signaling can enhance antioxidant capacity by regulating antioxidant enzyme activities and the expression of ROS-scavenging genes under abiotic stress conditions (Mo et al., 2024; Yu et al., 2023; Zhao et al., 2025).

In the present study, ABA seed priming increased SOD and POD activities and reduced MDA and H_2_O_2_ contents under low-temperature stress (**Figure 2**), indicating that ABA seed priming improved antioxidant capacity and alleviated oxidative damage. At the molecular level, ABA seed priming altered the expression of the examined SOD-related genes, including *SODB*, *SOD2-1*, *CCS*, and *SODCC1* (**Figure 3**). The gene expression responses were concentration- and gene-specific. T2 enhanced the expression of *SODB*, *SOD2-1*, and *CCS*, whereas *SODCC1* was downregulated under T2, T3, and T4. These results indicate that ABA seed priming did not uniformly activate all examined SOD-related genes, but differentially affected specific components of the SOD-associated antioxidant system.

Because only four SOD-related genes were examined, the present results provide limited molecular evidence for ABA-associated antioxidant regulation and cannot fully explain the broader ABA signaling network involved in low-temperature adaptation. Nevertheless, the coordinated increases in antioxidant enzyme activities, the gene-specific changes in selected SOD-related genes, and the reductions in H_2_O_2_ and MDA accumulation suggest that ABA seed priming may improve cold-stressed germination partly by enhancing ROS detoxification and reducing oxidative injury.

### 4.3 ABA seed priming is associated with altered endogenous ABA-GA status during cold-stressed germination

ABA and GA are key hormones regulating seed germination. GA promotes endosperm weakening, alpha-amylase synthesis, starch degradation, and embryo growth, thereby facilitating germination (Park et al., 2010). In contrast, ABA is generally associated with seed dormancy, stress signaling, and growth inhibition. Therefore, the balance between ABA and GA, rather than the absolute level of either hormone alone, is important for determining whether seeds remain dormant or initiate germination.

In the present study, low-temperature stress increased endogenous ABA content and decreased GA content, resulting in a higher ABA/GA ratio. Low-to-moderate ABA seed priming treatments, especially T2 and T3, were associated with reduced endogenous ABA content, increased GA content, and a lower ABA/GA ratio (**Figure 4**). These changes coincided with improved germination performance, increased alpha-amylase activity, enhanced soluble sugar accumulation, and higher respiratory rate.

The reduction of endogenous ABA after exogenous ABA seed priming may reflect feedback regulation of ABA homeostasis, such as changes in ABA biosynthesis or catabolism (Danieli et al., 2023; Zhang et al., 2023; Kim et al., 2025). However, ABA biosynthesis and catabolism genes were not examined in this study. Similarly, although GA content increased under appropriate ABA seed priming treatments, GA biosynthesis and catabolism genes such as *OsGA20ox*, *OsGA3ox*, and *OsGA2ox* were not measured. Therefore, the present results cannot demonstrate direct transcriptional regulation of ABA or GA metabolism by ABA seed priming.

Overall, these findings suggest that ABA seed priming was associated with altered endogenous ABA-GA status during germination under low-temperature stress. Low-to-moderate ABA concentrations may help coordinate stress adaptation and developmental progression, whereas excessive ABA may strengthen stress-related inhibition and reduce germination efficiency. However, the causal relationship among ABA seed priming, GA accumulation, reserve mobilization, and germination requires further validation using hormone metabolism gene analysis, GA inhibitor treatments, and rescue experiments.

### 4.4 Potential involvement of ABA-related signaling requires further validation

ABA-mediated stress responses are generally regulated through the PYR/PYL/RCAR-PP2C-SnRK2 signaling pathway. Under stress conditions, ABA is perceived by PYR/PYL/RCAR receptors, which inhibit PP2C phosphatases and release SnRK2 protein kinases from repression. Activated SnRK2s phosphorylate downstream transcription factors and stress-responsive proteins, leading to changes in gene expression, osmotic adjustment, ROS metabolism, and stress tolerance (Mo et al., 2024; Zhao et al., 2025).

In the present study, low-to-moderate ABA seed priming enhanced antioxidant enzyme activities, increased soluble sugar content, and was associated with altered endogenous ABA-GA status under low-temperature stress (**Figures 2**-**4**). These physiological changes are consistent with downstream responses commonly associated with ABA-related stress signaling. ABA signaling may also interact with cold-responsive transcription factors, such as CBF/DREB, thereby contributing to cold adaptation (Mo et al., 2024; Zhao et al., 2025; Song et al., 2021).

However, the present study did not directly examine PYR/PYL receptors, PP2Cs, SnRK2s, ABF/AREB transcription factors, or CBF/DREB genes. Therefore, the involvement of ABA signaling remains inferential rather than directly demonstrated. The observed changes in antioxidant enzyme activities, soluble sugar accumulation, hormone contents, and selected SOD-related gene expression suggest possible downstream responses associated with ABA seed priming, but the precise signaling pathway requires further molecular validation.

### 4.5 Persistence of ABA seed priming effects into seedling growth

Seed priming can induce physiological and molecular adjustments during imbibition and germination that persist into later developmental stages. Such priming effects may involve improved reserve mobilization, enhanced antioxidant capacity, hormonal reprogramming, and stress memory, enabling seedlings to respond more effectively to subsequent stress conditions (Chen et al., 2023; Monajjem et al., 2023; Wang et al., 2016).

In the present study, ABA seed priming not only improved germination performance under low-temperature stress but also enhanced seedling quality and antioxidant enzyme activities at the three-leaf stage (**Table 4**; **Figure 5**). ABA seed priming treatments consistently improved root growth during both germination and seedling development, suggesting that the beneficial effects established during germination may persist during subsequent growth. Stronger root development may facilitate water and nutrient acquisition, thereby improving seedling establishment and adaptation to low-temperature environments.

The correlation analysis further supported this interpretation. Positive relationships between germination-stage physiological traits and seedling-stage growth performance indicate that early improvements in reserve mobilization, antioxidant defense, and hormonal status may contribute to subsequent growth advantages. In contrast, MDA and H2O2 contents were negatively associated with seedling performance, suggesting that reduced oxidative damage during germination may facilitate later seedling development. Collectively, these findings suggest that ABA seed priming established during germination may contribute to improved early seedling establishment under low-temperature stress in direct-seeded rice.

### 4.6 Concentration-dependent trends of ABA seed priming

The effects of ABA on seed germination and stress adaptation are highly concentration-dependent. In the present study, concentration-dependent trends suggested that low-to-moderate ABA concentrations improved germination performance, antioxidant capacity, and endogenous ABA-GA status under low-temperature stress, whereas excessive ABA weakened these beneficial effects.

Among the tested concentrations, 40 μmol L^−1^ ABA showed the most favorable overall performance, including higher germination performance, enhanced SOD activity, reduced MDA accumulation, and a lower ABA/GA ratio. These results indicate that 40 μmol L^−1^ ABA was the most favorable concentration for improving low-temperature germination performance in direct-seeded rice under the present experimental conditions.

The observed concentration-dependent response may reflect the dual role of ABA in stress adaptation and growth regulation. Moderate ABA levels may activate protective mechanisms while maintaining growth-related metabolism, whereas excessive ABA may enhance stress signaling at the expense of germination and seedling development. Therefore, the beneficial effects of ABA seed priming depend on maintaining an appropriate concentration range, with 40 μmol L^−1^ showing the best balance between stress protection and growth performance among the tested concentrations.

### 4.7 Limitations and future perspectives

Although this study provides physiological and limited molecular evidence that ABA seed priming improves germination and early seedling establishment under low-temperature stress in direct-seeded rice, several limitations should be acknowledged. Only one japonica rice cultivar, Changbai 9, was used. Different rice cultivars vary in genetic background, endogenous hormone regulation, reserve mobilization efficiency, antioxidant capacity, and chilling tolerance. Therefore, the magnitude and direction of ABA-induced responses may differ across genotypes (Danieli et al., 2023; Wang et al., 2016). Future studies should validate these findings in multiple japonica and indica cultivars to assess the broader applicability of ABA seed priming. ABA seed priming under normal-temperature conditions was not included. Therefore, this study cannot fully distinguish general ABA priming effects from low-temperature-specific effects. Including an ABA-primed normal-temperature treatment in future experiments would help clarify whether the beneficial effects of ABA seed priming are specific to low-temperature stress or also occur under favorable germination conditions.

Although endogenous ABA and GA contents were measured, hormone metabolism-related genes were not examined. In particular, GA biosynthesis and catabolism genes such as *OsGA20ox*, *OsGA3ox*, and *OsGA2ox* were not analyzed. Therefore, the present results cannot determine whether ABA seed priming directly regulates GA metabolism at the transcriptional level. Studies should combine hormone quantification with gene expression analysis of ABA and GA metabolic pathways. Fourth, GA inhibitor or rescue experiments were not performed. For example, paclobutrazol treatment or GA rescue experiments would be useful for determining whether GA accumulation is causally involved in ABA-associated alpha-amylase activity, reserve mobilization, and germination under low-temperature stress. ROS accumulation was evaluated using biochemical H_2_O_2_ and MDA measurements, but *in situ* ROS visualization using DAB or NBT staining was not conducted. Therefore, the spatial distribution of ROS in germinating seeds remains unclear. Future studies combining biochemical assays with histochemical staining would provide stronger evidence for ABA-associated ROS regulation. Molecular analyses were limited to four SOD-related genes, namely *SODB*, *SOD2-1*, *CCS*, and *SODCC1*. This provides only partial insight into antioxidant-related gene regulation. The study did not examine core components of the PYR/PYL-PP2C-SnRK2 ABA signaling pathway or cold-responsive transcription factors such as CBF/DREB. Thus, while the observed physiological changes are consistent with ABA-associated stress responses, the molecular basis remains incompletely resolved. Finally, the experimental design focused on controlled conditions. Environmental variability in field settings, such as fluctuating temperatures, soil moisture, and nutrient availability, may influence ABA seed priming responses. Comprehensive transcriptomic, metabolomic, hormone-profiling, and field-based studies are needed to clarify the broader regulatory networks underlying ABA seed priming-induced low-temperature adaptation.

In summary, this study suggests that ABA seed priming can alleviate low-temperature-induced inhibition of germination and improve early seedling establishment in direct-seeded rice. These effects were associated with improved reserve mobilization, enhanced antioxidant capacity, reduced oxidative damage, and altered endogenous ABA-GA status. However, direct regulation of GA metabolism and ABA signaling was not demonstrated in this study. Further work is required to validate the underlying molecular mechanisms and to generalize these findings across genotypes and field conditions.

## 5 Conclusion

In conclusion, ABA seed priming alleviated low-temperature-induced inhibition of germination and improved early seedling establishment in direct-seeded rice. Low-to-moderate ABA concentrations were associated with enhanced reserve mobilization and energy supply, as indicated by increased alpha-amylase activity, soluble sugar accumulation, and respiratory metabolism during germination under low-temperature stress. ABA seed priming also improved antioxidant capacity by increasing SOD and POD activities and reducing H_2_O_2_ and MDA accumulation. Expression analysis of selected SOD-related genes showed concentration- and gene-specific responses, indicating that ABA seed priming did not uniformly activate all examined SOD-related genes. In addition, ABA seed priming was associated with altered endogenous ABA-GA status, including a reduced ABA/GA ratio, which may contribute to the coordination between stress adaptation and germination progression. The correlation analysis and seedling-stage responses further suggested that the beneficial effects established during germination may persist into later developmental stages, contributing to improved seedling growth under low-temperature stress. Among the tested concentrations, 40 umol L^-1^ ABA showed the most favorable overall performance, indicating that the effect of ABA seed priming was concentration-dependent within the tested range. Overall, these findings suggest that ABA seed priming improves cold-stressed germination and early seedling establishment mainly through physiological adjustments involving reserve mobilization, antioxidant defense, oxidative damage mitigation, and endogenous hormonal status. However, direct regulation of GA metabolism, ABA signaling, and broader molecular networks was not demonstrated in this study. Further studies integrating ABA signaling components, GA metabolism-related genes, ROS visualization, transcriptomic analyses, inhibitor or rescue experiments, and multiple rice cultivars are needed to clarify the regulatory mechanisms underlying ABA seed priming-induced low-temperature adaptation in direct-seeded rice.

## Data Availability

The original contributions presented in the study are included in the article/**Supplementary Material**. Further inquiries can be directed to the corresponding authors.
