# Supplementary material for: ABA seed priming alleviates low-temperature-induced inhibition
Source: Front Plant Sci. 2026 Jul 9;17:1866445. doi: 10.3389/fpls.2026.1866445 (PMC13392933; doi:10.3389/fpls.2026.1866445)
Supplement: Supplementary file 1 [file DataSheet1.docx]

Supplementary Material

Table S1 Temperature and light setting for climate-control chamber

| Time | Light intensity | Temperature |
| --- | --- | --- |
| 6:00-10:00 | 5000 | 20 |
| 10:00-15:00 | 10000 | 20 |
| 10:00-18:00 | 5000 | 20 |
| 18:00-6:00 | 0 | 15 |

Table S2 Real-time PCR reaction system

| Component | Volume (μL) |
| --- | --- |
| SYBR Premix Ex TaqⅡ (Tli RNaseH Plus) (2×) | 10 μL |
| PCR Forward Primer (10μM) | 0.8 μL |
| PCR Revase Primer (10μM) | 0.8 μL |
| Rox Reference Dye (50×) | 0.4 μL |
| DNA template | 2 μL |
| Sterilized water | 6 μL |
| Total | 20 μL |

Table S3 Gene-specific primers used in real time PCR analysis

| Gene name | Forward prim | Revase prime |
| --- | --- | --- |
| *SODB* | CGAAGGTATTCAAAAGTCGTGG | AGTTCAACTGTCCTCTTGCTAA |
| *SOD2-1* | TGGCTTCCACATCCACTCCTT | CAACAACTGCCCTTCCCAATAT |
| *SODCC1* | ATCCTGATGATCTTGGAAAGGG | AAAACACATAGTTCATTGGGCG |
| *CCS* | GGTAAACACGGATGGTCAATAAA | TGTCCTCAGTGGCATACAACG |


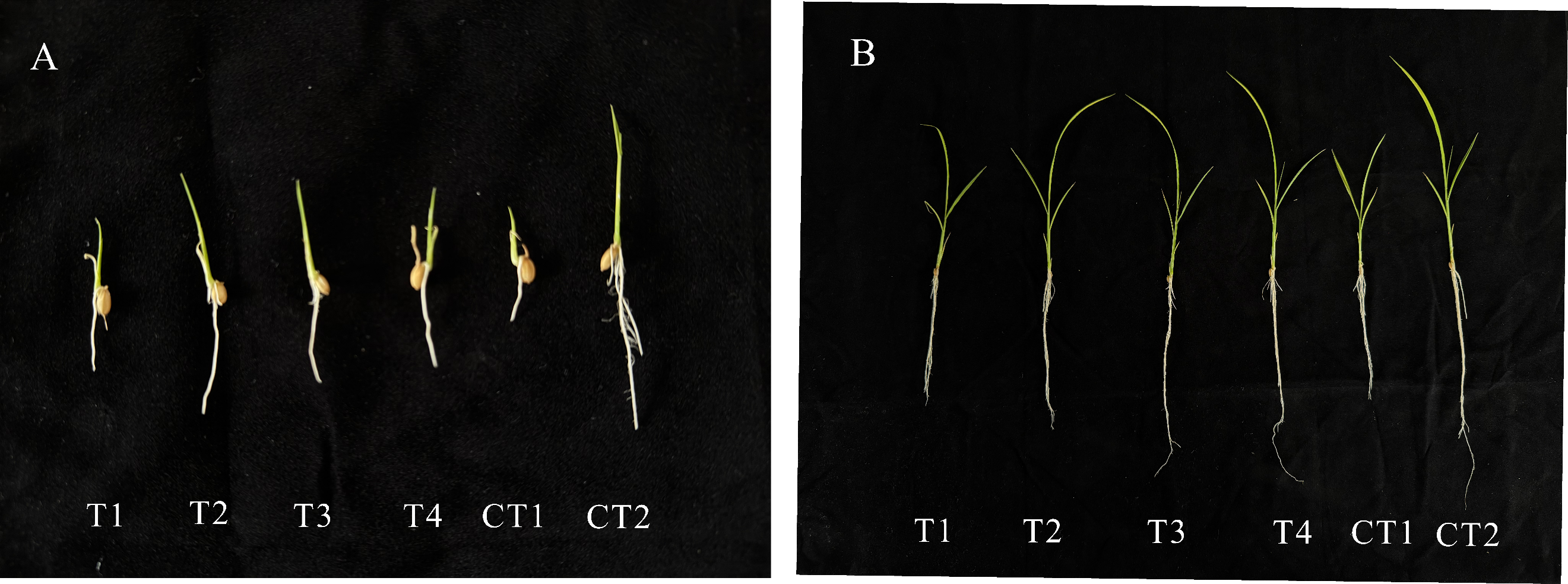


Figure S1. 7 days after germination (A) and the three-leaf stage (B).
